# Development and validation of a simplified algorithm for neonatal gestational age assessment – protocol for the Alliance for Maternal Newborn Health Improvement (AMANHI) prospective cohort study

**DOI:** 10.7189/jogh.07.021201

**Published:** 2017-12

**Authors:** AMANHI (Alliance for Maternal and Newborn Health Improvement), Abdullah Baqui, Parvez Ahmed, Sushil Kanta Dasgupta, Nazma Begum, Mahmoodur Rahman, Nasreen Islam, Mohammad Quaiyum, Betty Kirkwood, Karen Edmond, Caitlin Shannon, Samuel Newton, Lisa Hurt, Fyezah Jehan, Imran Nisar, Atiya Hussain, Naila Nadeem, Muhammad Ilyas, Anita Zaidi, Sunil Sazawal, Saikat Deb, Arup Dutta, Usha Dhingra, Said Moh’d Ali, Davidson H. Hamer, Katherine EA Semrau, Marina Straszak–Suri, Caroline Grogan, Godfrey Bemba, Anne CC Lee, Blair J Wylie, Alexander Manu, Sachiyo Yoshida, Rajiv Bahl

**Affiliations:** 1 AMANHI Gestational Age Study Group, Bangladesh (Sylhet); 2 AMANHI Gestational Age Study Group, Ghana; 3 AMANHI Gestational Age Study Group, Pakistan (Karachi); 4 AMANHI Gestational Age Study Group, Tanzania (Pemba); 5 AMANHI Gestational Age Study Group, Zambia; 6 Brigham & Women’s Hospital, Massachusetts General Hospital, Boston, Massachusetts, USA; 7 World Health Organization (MCA/MRD), Geneva, Switzerland

## Abstract

**Objective:**

The objective of the Alliance for Maternal and Newborn Health Improvement (AMANHI) gestational age study is to develop and validate a programmatically feasible and simple approach to accurately assess gestational age of babies after they are born. The study will provide accurate, population–based rates of preterm birth in different settings and quantify the risks of neonatal mortality and morbidity by gestational age and birth weight in five South Asian and sub–Saharan African sites.

**Methods:**

This study used on–going population–based cohort studies to recruit pregnant women early in pregnancy (<20 weeks) for a dating ultrasound scan. Implementation is harmonised across sites in Ghana, Tanzania, Zambia, Bangladesh and Pakistan with uniform protocols and standard operating procedures. Women whose pregnancies are confirmed to be between 8 to 19 completed weeks of gestation are enrolled into the study. These women are followed up to collect socio–demographic and morbidity data during the pregnancy. When they deliver, trained research assistants visit women within 72 hours to assess the baby for gestational maturity. They assess for neuromuscular and physical characteristics selected from the Ballard and Dubowitz maturation assessment scales. They also measure newborn anthropometry and assess feeding maturity of the babies. Computer machine learning techniques will be used to identify the most parsimonious group of signs that correctly predict gestational age compared to the early ultrasound date (the gold standard). This gestational age will be used to categorize babies into term, late preterm and early preterm groups. Further, the ultrasound–based gestational age will be used to calculate population–based rates of preterm birth.

**Importance of the study:**

The AMANHI gestational age study will make substantial contribution to improve identification of preterm babies by frontline health workers in low– and middle– income countries using simple evaluations. The study will provide accurate preterm birth estimates. This new information will be crucial to planning and delivery of interventions for improving preterm birth outcomes, particularly in South Asia and sub–Saharan Africa.

Accurately identifying preterm births around the time of delivery increases opportunities for using targeted high impact interventions to reduce mortality and morbidity but requires that gestational age at birth be known. Preterm birth is a global challenge affecting both developed and developing countries [1,2]. It contributes annually to approximately 35% of neonatal and 75% of perinatal mortality worldwide [3–5]. In addition, survivors suffer long–term respiratory, gastrointestinal and neurodevelopmental morbidities with consequences on health, growth, psychosocial functioning and economic viability in later life [6–8]. The greatest burden of mortality and morbidity from preterm births occur in low and middle income settings (LMICs) [9]. In 2005, almost 13 million children were born preterm and 85% of these occurred in Africa and Asia [10].

Interventions that are feasible to implement and have the potential to prevent or help manage preterm birth complications in LMICs exist, but their effectiveness has not been rigorously evaluated [11]. The critical step to deploying such interventions is identification of preterm births. In high–income settings, early pregnancy ultrasound is used to enhance accurate gestational age estimation. Preterm births are therefore anticipated right at the onset of labour and interventions such as antenatal corticosteroids are administered to improve outcomes. In contrast, pregnancy ultrasound for assessing gestational age is often not available or affordable to women in LMICs [12]. Pregnant women also seek care quite late; by which time most ultrasound scans do not yield reliable gestational age estimates. To help frontline health workers identify preterm babies for targeted interventions, simpler methods of gestational age assessment are needed.

One option is to train them to identify preterm babies immediately after they are born. The Ballard [13,14] and Dubowitz [15,16] scoring systems use physical and neuromuscular signs to estimate newborn gestational maturity after birth. Whilst they are potentially feasible to implement in LMICs, they are rarely used at scale partly because they have not been rigorously evaluated in comparison with a reliable “gold standard” or because they include too many component signs that are difficult to assess on all babies. For instance, five [14,17–20] of six studies that evaluated the accuracy of these scores compared them with women’s last menstrual period (LMP) that is itself not reliable for gestational age assessment. Meanwhile, portable ultrasound machines are now available, relatively cheap and can be deployed to allow improved gestational age assessment at population level or within research settings in LMICs. In addition, advances in computer programming and “machine learning” methods enable simpler algorithms with equivalent validity to be derived from complex patterns and inter–relationships between signs.

The Alliance for Maternal and Newborn Health Improvement (AMANHI) gestational age study is being implemented in five countries in South Asia and sub–Saharan Africa. It aims to develop and validate simple, programmatically feasible methods (using non–clinically–trained research workers) to accurately assess gestational age of babies at the population level in comparison to early pregnancy ultrasound (gold standard). The study will also use these accurate gestational ages to evaluate the risk of mortality and morbidity, by gestational age, among neonates. This manuscript describes the protocol for the harmonized implementation of the study.

## METHODOLOGY

### Study design

The AMANHI gestational age study is a multi–centre, population–based prospective development and evaluation of the diagnostic accuracy of simple methods for gestational age assessment (including reported LMP, physical, neuromuscular, feeding assessments and anthropometry). These will be compared with gestational age from early pregnancy ultrasound scans. The Maternal, Newborn, Child and Adolescent Health department of the World Health Organization (WHO/MCA) is coordinating the study.

### Objectives

The objective of the AMANHI gestational age study is to develop and validate a programmatically feasible and simple approach to accurately assess the gestational age of babies after they are born. The study will provide accurate, population–based rates of preterm birth in different settings and quantify the risks of (i) neonatal mortality by gestational age and birthweight, and (ii) maternal and neonatal morbidity by gestational age from population–based pregnancy cohorts.

### Study settings

Five sites in South Asia (Sylhet, Bangladesh and Karachi, Pakistan) and sub–Saharan Africa (Kintampo, Ghana; Pemba, Tanzania; and Southern Province, Zambia) are using harmonized protocols to implement the AMANHI gestational age study. Across the sites, study populations are predominantly rural with low literacy levels. Health facilities of various types provide a range of services from basic to comprehensive emergency obstetric and newborn care. All sites, except Zambia, have an on–going one to three–monthly community–based pregnancy surveillance in which trained fieldworkers conduct home visits to all women of reproductive age to identify pregnancies for enrolment and follow–up until the end of pregnancy. In Zambia, the study is recruiting pregnant women at antenatal clinics.

### Identifying eligible pregnancies and obtaining consent

**Eligibility.** Trained fieldworkers use a variety of methods to identify pregnant women for the study. Women either directly report their pregnancies or the fieldworkers elicit missed periods from women’s reported dates of their LMP. They also conduct pregnancy tests, if required, to confirm pregnancies. In Zambia, over 96% of all pregnant women attend antenatal care (ANC) clinics so study participants are recruited from these clinics. The study enrols women only if their pregnancies are between 8–19 completed weeks by ultrasound examination. This window ensures that pregnancies are early enough to give accurate ultrasound gestational age estimates but also excludes very early pregnancy losses due to genetic or chromosomal abnormalities. Enrolled women should also be willing to be available for the gestational age assessment to be conducted on their babies within 72 hours after birth.

**Consenting.** Fieldworkers consent pregnant women, in their local or preferred languages, to undergo a screening ultrasound examination to establish gestational age and subsequently enrol women if eligibility is confirmed (pregnancies between 8–19 weeks’ gestation). Consent for additional procedures varies slightly across sites. In Ghana, for instance, fieldworkers obtain initial consent for the screening ultrasound. They only consent eligible women after scan and again before the gestational age assessment when the baby is born. In other sites, women are comprehensively consented at the initial contact to cover the scan and follow–up. In Zambia in case the pregnant woman is a “minor” between 15 years to 18 years, assent is first obtained from women themselves before their legal guardians provide written informed consent for their participation.

### Harmonization of implementation

Uniform protocols are being used across sites to conduct the screening ultrasound scan, pregnancy follow–up, newborn gestational age assessments and quality assurance. The principal investigators from the sites, WHO/MCA team and selected global newborn experts supported the development of these protocols for use at all sites. Centralised training sessions were organised for investigators from the sites to ensure harmonised implementation.

#### Standard operating procedures

**Figure 1:**
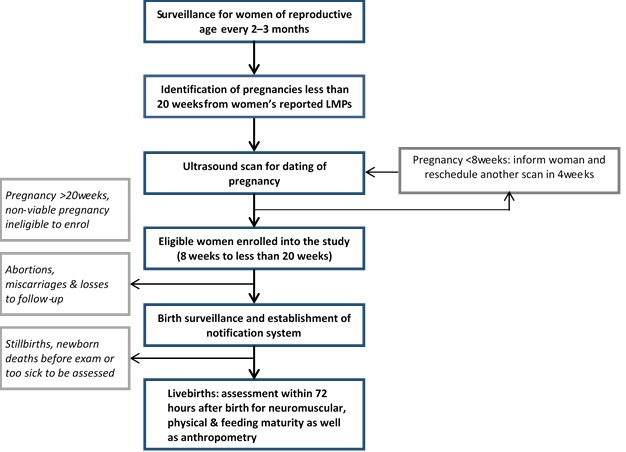
Flow diagram showing overview of the AMANHI gestational age study.

Principal investigators from the sites developed generic standard operating procedures (SOP) for implementing the study. This document outlines the strategy for implementation as illustrated in **Figure 1**. In summary, it specifies that the gold standard gestational age will be derived from an early pregnancy ultrasound scan (<20 weeks) conducted as part of the study by trained study staff. The study enrols pregnant women during the gestational age window of 8 to 19 completed weeks. Biometric parameters measured include fetal crown–rump length (CRL), bi–parietal diameter (BPD) and femur length (FL) as appropriate. Trained non–clinical workers conduct the gestational age assessment of newborns. With this cadre of workers, the AMANHI gestational age study ensures that minimally trained frontline health workers can implement postnatal gestational age assessment, at scale. A common manual for implementation and core variable table have been developed, which contains modules on the standards for the dating ultrasound, newborn gestational age assessment and data collected during follow–up home visits.

#### Centralised standardised training and validation of trainers for newborn assessment

AMANHI organised a 3–day training of trainers in Sylhet (Bangladesh) for two physician participants from each site, using the AMANHI gestational age study manual. The training, coordinated by WHO/MCA, aimed to standardize these participants for harmonised implementation across sites. Two experts, a WHO Medical Officer in newborn health (AM) and a paediatrician specialist in newborn maturity assessment (ACL) facilitated the training. It involved theoretical instructions, videos and practice sessions. After showing videos of the assessment, the facilitators demonstrated the assessment modalities to the participants on mannequins, with emphasis on the assessment principles. Participants in turn practiced on these mannequins to acquaint themselves with the assessment modalities. The last two days of training involved practice at the paediatric ward of the Osmani Medical College in Sylhet. The trainers again demonstrated assessments to participants using newborn babies on the wards. They supervised the participants to take turns, practicing initially with term and later with preterm babies. Participants were closely supervised and provided additional support to ensure that they became confident and proficient in the assessments. For the proficiency validation and standardization, each participant independently assessed five or more babies and recorded their findings. Trained facilitators also independently assessed these same babies and the findings were compared with the participants. Participants were only certified as having attained mastery for any particular sign if their assessment findings did not vary by more than one point from the findings from the independent assessment by the facilitators.

#### Ultrasonography for accurately dating pregnancies (gold standard data)

The SOP for the AMANHI gestational age study specifies standardized procedures for measuring fetal biometric parameters, and was developed in consultation with a maternal–fetal medicine specialist (BJW). Scanning is strictly trans–abdominal. When a potential participant comes to a scanning centre to have her pregnancy date assessed, the choice of which biometric parameter to measure depends on the estimated gestational age; this was to ensure that gestational ages estimated from these parameters have the highest precision (**Table 1**). AMANHI measures only the crown–rump length (CRL) if pregnancy is less than 14 weeks, both bi–parietal diameter (BPD) and femur length (FL) if more than 14 weeks and all three if within the 14th week. Three measurements are independently taken and recorded for each parameter. Pregnancies that are less than 8 weeks are re–scheduled for repeat scans after four weeks. Women with pregnancies at 20 weeks or more are counselled to continue routine antenatal clinic attendance but are not enrolled into the AMANHI gestational age study. Major abnormalities or intra–uterine fetal deaths are promptly referred to health facilities for appropriate management.

**Table 1:** Choice of fetal biometric parameter by estimated gestational age in AMANHI

Gestational age of fetus	What biometric parameter to measure
8 weeks 0 days – 13 weeks 6 days	Crown–rump length only
14 weeks 0 days – 14 weeks 6 days	Crown–rump length, bi–parietal diameter & femur length
15 weeks 0 days and beyond	Bi–parietal diameter and femur length

**Quality control.** The team developed a common checklist, in consultation with the MFM expert and with adaptations from other international ultrasound quality guidelines [21], which specifies the minimum acceptable quality standards for measuring each biometric parameter (**Table 2**). This checklist serves as a reference (and harmonization) document for the sonographers at the sites as well as a yardstick for independent assessment of image quality and includes guidance on appropriate image zooming, obtaining the correct plane and placement of callipers.

**Table 2:** Indicators for ensuring optimal quality of ultrasound images for each biometric parameter

Quality modality	Biometric parameter		
**Crown rump length**	**Bi–parietal diameter**	**Femur length**
**Zooming**	Good magnification (50% of image size)	Good magnification (50% of image size)	Good magnification (50% of image size)
**Frozen in correct plane**	Neutral position–not hyperflexed or hyperextended	Skull is oval and bone visible throughout	Femur imaged side–to–side on screen
	Fetus horizontal (side–to–side on screen)	Thalamus is visible	Only one bone in this portion of extremity
	Full extent of crown visible	Skull side to side on screen	Upper femur measured
	Full extent of rump visible		Full extent of femur visualized (solid straight line)
**Caliper placement**	Crown caliper at edge of head	Calipers placed perpendicular to long axis of skull	Calipers placed at edge of echogenic bone (outer to outer)
	Rump caliper placement at lower spine	Top caliper placed on outer portion of the skull	Secondary ossification centres not measured
		Bottom caliper placed on inner portion of skull	

Key quality control measures include:

- The MFM expert independently reviews all images taken for 20 among the first 50 study participants recruited in each site and provides direct feedback on each individual image and the overall performance of sonographers. She also makes recommendations on how to further improve image quality.
- Checking images throughout the duration of enrolment;
  − Images taken for 10% of participants are randomly selected and sent to a specialist sonographer (local to each site but independent of the AMANHI study team) for review. This independent sonographer provides direct feedback to inform refresher–training needs of study sonographers within each site.
  − Images taken for 5% of participants are randomly selected and sent for central review and validation by the MFM expert (BJW).

#### Morbidity and birth outcome surveillance

Trained surveillance fieldworkers visit women in their homes, immediately after enrolment, to collect baseline socio–demographic, medical and obstetric histories. They make three antenatal visits at 24–28 weeks, 32–36 weeks and after 38 weeks to collect data on morbidity and mortality during the pregnancy and pregnancy outcomes using the common core variable table. Women’s blood pressure is measured and their urine is tested for proteins. In some sites, women’s symphysis–fundal height is also measured. During these visits, fieldworkers establish a birth notification system so that families will notify the study whenever the pregnancy ends – irrespective of the outcome. For live births, immediately after the notification, research assistants are dispatched to reach babies and conduct AMANHI gestational age assessment within 72 hours of birth. The surveillance fieldworkers also increase their frequency of home visits to all women whose pregnancies are 28 weeks or older and leave their phone numbers for families to contact them whenever the pregnancy ends. When any pregnancy ends, additional home visits are made to ascertain the status of newborns and mothers at the end of the first 28 and 42 to 60 days, days respectively. For every maternal, fetal or neonatal death, a verbal autopsy form is completed to obtain detailed information on the circumstances leading to the death; causes of death are assigned by trained and AMANHI–accredited physicians. The accreditation process involves training for harmonized assignment of causes of deaths and passing of a centralised standardised test conducted by the WHO [22].

#### Newborn assessment

**Assessors.** Non–clinician field workers (at supervisory grade and with ≥10 years formal education) were trained and standardized to conduct the AMANHI newborn gestational age assessments. These assessors are blinded to babies’ ultrasound–estimated gestational ages.

**Inclusion and exclusion criteria.** The assessors screen for newborn illnesses before conducting the newborn assessment. Families are engaged throughout the assessment process. The assessors ask to know about the families’ perception on the general health of the newborn. If babies are reportedly unwell, they use the newborn assessment module of WHO’s integrated management of childhood illness (IMCI) guidelines to assess severity. Babies with severe illness are excluded and referred to health facilities for care. Even if the family thinks the baby is well but the assessors consider them unwell, they also refer to health facilities for care. In general, assessors advise care–seeking at health facilities even when the IMCI algorithm shows the newborn is not ill but the family thinks otherwise.

**Assessment modalities.** **Table 3** shows signs being assessed for in the AMANHI gestational age study. These signs were selected based on a systematic literature review for evidence of their use in assessing newborn gestational maturity and a pragmatic consideration of how easily they could be assessed by frontline health workers. There are six neuromuscular signs which test passive flexor tone or flexibility and 10 physical signs. The physical and neuromuscular signs were selected from the original Ballard score [14]. Each sign relates to a particular body part (eg, skin), under which are sub–categories such as colour, opacity, texture, etc. The separate sub–categories were adapted from the Dubowitz score [15,16] because they are separated and relatively easier to differentiate. After discussions with Dubowitz (personal communication), ankle dorsiflexion was added to the neuromuscular signs as a measure of joint flexibility. The “square window” sign was dropped due to parental concerns during piloting.

**Table 3:** Assessment modalities with acceptable precision (for anthropometric measurements)

Assessment	Signs	Precision (if applicable)
Neuro–muscular signs	▪ Posture ▪ Arm recoil ▪ Scarf sign ▪ Popliteal angle ▪ Heel–to–ear test ▪ Ankle dorsiflexion	
Physical signs	▪ **Skin**: colour, texture, opacity and presence of lanugo ▪ **Ear**: shape and recoil ▪ **Breast**: nipple–areola development ▪ **Male genitalia**: testes and scrotum ▪ **Female genitalia**: labia and clitoris ▪ **Foot**: plantar creases	
Anthropometry	▪ Head circumference (cm)	±0.5 cm
	▪ Chest circumference (cm)	±0.5 cm
	▪ Breast bud diameter (mm)	±1 mm OR ±0.1 cm
	▪ Mid–upper arm circumference (cm)	±2 mm OR ±0.2 cm
	▪ Foot length (mm)	±2 mm OR ±0.2 cm
	▪ Infant length (cm)	±0.5 cm
	▪ Symphysis–fundal height (cm)	±1.0 cm
	▪ Weight (g)	±40 g if digital scale & 100 g if suspension scale
Feeding	▪ Check whether the infant is able to attach well to the breast by observing four signs: mouth open; lower lip everted; chin touching the breast below; greater part of the areola in mouth and more areola seen above than below.	
	▪ Observation: Suckling behaviour of the baby at the breast – deep, slow continuous suck with swallowing in between or shallow quick sucks.	
	▪ Observe: Duration the infant was able to stay attached to the breast continuously during the feed?	
	▪ Observation: The longest continuous burst of suckling (number of sucks)	
	▪ While observing breastfeeding, how many times did the baby suck before swallowing?	

Seven anthropometric measures (head, chest and mid–upper arm circumference, infant length, birth weight, breast–bud diameter, infant foot length and (optionally) women’s symphysis–fundal height) in pregnancy are taken. Foot length (right foot) is measured from base of the heel to the tip of the halux, based on methods proposed by Marchant et al [23]. For all the measurements, two are taken independently and if their difference exceeds a set acceptable margin of error (**Table 3**), a third measurement is taken. All three measurements are recorded. Five signs of feeding maturity, adapted from WHO’s infant feeding assessment guide with additions (on suckling bursts and suck–to–swallow ratios) from the Nvquist [24] preterm feeding questionnaire are also assessed. Assessors record their findings on a standard AMANHI form.

#### Supervision of assessment

This involves direct observation, scheduled and un–scheduled visits to study assessors. Study coordinators supervise assessors on the field. They directly observe 5% of assessments conducted by the Research Assistants in the communities as part of the supervision. These 5% selected observations are spread over the entire duration of the study to ensure assessors’ skills are being maintained. The coordinators have been purposively selected from a pool of supervisors who have performed excellently in previous engagements as supervisors in the sites. Their qualifications range from post–secondary to university graduates. As well as their extensive previous experience in field research, they were provided additional training in the conduct of the gestational age assessment and then on how to supervise and provide feedback to the assessors. The coordinators also make scheduled and unscheduled visits to assessors in the field to observe their work. In Tanzania and Ghana, geographical information systems are used to locate assessors for these supervisory visits. After each supervisory visit session, feedback on performance is provided to the assessor.

In some sites including Ghana, a pilot implementation phase followed the assessors’ training during which the study clinician/trainer conducted intensive supervision of assessors and directly observed five or more of their newborn assessments. That phase was also used to train field coordinators on the practicality of supervising the assessors.

#### Professional clinician validation of assessment

In order to validate the assessment by the non–clinicians, a trained professional clinician/trainer independently validates a random 5–15% sub–sample of assessments conducted by the assessors. In some sites, the process is centrally coordinated and an automated system or a study coordinator selects these babies, informs the physician of the deliveries and provides them with women’s location to facilitate the validation assessment. The physician visits the woman at the place of delivery, also within 72 hours, to independently assess babies using the AMANHI assessment forms.

#### Study materials/equipment

The AMANHI gestational age study teams procured uniform equipment and materials for the study across sites. Where materials could not be procured from a common source, the desired quality was specified in order to provide comparable error margins. Weighing scales, non–stretchable measuring tapes, transparent rulers and infantometers were procured and are used for the assessment across sites.

### Supportive activities

In all the sites, the AMANHI study built on existing long–standing relationship of trust and mutual respect between investigators, communities and health facilities. They held meetings with community members (and their leaders) to introduce the study and to enhance acceptability of assessments. Separate orientation sessions were organised in health facilities to solicit their support and discuss the potential impact of the AMANHI gestational age study on facility workload. These included discussions on referral of complicated pregnancies for appropriate management. In some sites, a separate training was conducted for facility staff to assess selected physical signs on babies considered too sick to be assessed by the non–clinician assessors.

### Sample size estimation

Previously pooled data from the participating sites showed that approximately 10% of all births were preterm. In the AMANHI gestational age study, it was assumed that the simpler methods will detect this prevalence with ±5% (absolute) precision and achieve 80% sensitivity and specificity in comparison to the early pregnancy ultrasound gestational age estimate. With an additional 1.5–fold adjustment for design effect (to account for contextual differences between study sites) and 20% attrition rate (due to abortions/miscarriages, stillbirths, early neonatal deaths and losses to follow–up) [25], a minimum of 5740 pregnant women will be required to be enrolled. Machine learning approaches use separate pools of data from the same population for the identification of the algorithm (two thirds of total) and for the validation of this algorithm (one third of total). This increases the total targeted sample size to 8610, 5740 for machine learning and 2870 for validation. **Table 4** shows proposed sample size contributions from each site.

**Table 4:** Study sites and contribution to the overall study sample

Study sites	Minimum sample size contribution from study sites
Bangladesh (Sylhet)	2200
Ghana (Kintampo)	1000
Pakistan (Karachi)	2200
Tanzania (Pemba)	2200
Zambia (Southern Province, Zambia)	1000

### Data processing and management

All data are double–entered by two independent clerks at all sites except in Zambia, where they are scanned directly using TeleForms software (Hewlett Packard, Sunnyvale, CA). Principal investigators in the sites have excellent track records for management of large volumes of data with stringent protocols. They are locally responsible for maintaining the data quality in their sites, running range and consistency checks and conducting periodic reviews of distributions of the responses to identify outliers in the data to address promptly. The WHO/MCA also developed a data quality checking programme to be applied on all data to supplement the routine checks conducted by individual sites. Any inconsistencies were flagged and resolved in consultation with data managers and investigators from the sites.

### Plan for data analysis

The pooled data from the study sites will be split into equal thirds. Computer machine learning techniques will be employed on two–thirds of the data to find a group of signs that accurately and independently predicts gestational age or identifies preterm babies <34 weeks and/or <37 weeks compared to ultrasound dates (gold standard). The difference in gestational age estimates (in completed weeks) between the gold standard and the simpler methods (LMP and/or newborn assessments) will be plotted against the mean gestational ages (of gold standard and simplified method). Any identified algorithm that meets the requisite validity criteria will then be applied to the remaining one–third of data to assess validity (sensitivity, specificity and receiver operating characteristic curves).

Predicted gestational ages from the new algorithm will be used to categorize babies into those <34 weeks, 34–36weeks and those ≥37 weeks or a dichotomy of preterm (<37 weeks) vs term. Chance–corrected agreement and kappa statistics will be generated between these categories and those obtained from gestational age cut–offs based on ultrasound dating and interpreted using Landis and Koch [26] criteria. Regression models will also be fitted to determine association between gestational age and adverse maternal and neonatal outcomes. Analyses will be done using Stata® Version 14.0 (StataCorp., Tx, USA) and other specialized software.

### Ethical considerations

All study protocols have been approved by ethical review committees of the WHO and appropriate institutions in each of the participating sites.

### Dissemination of study findings

Study findings will be shared promptly with the Technical Steering Committee comprising the WHO/MCA coordinating team, the principal investigators from the five sites and the Bill & Melinda Gates Foundation. Local dissemination meetings will be held with community members, policy–makers, health managers and administrators in all sites. A detailed implementation evaluation report, including lessons learned will be shared with the WHO and Gates Foundation. Policy briefs on outcomes will be disseminated nationally and internationally to relevant policy and donor organisations. Study findings will also be disseminated in scientific/technical forums and in peer–reviewed journals.

## CONTRIBUTION OF THE AMANHI GESTATIONAL AGE STUDY TO PRETERM BIRTH IDENTIFICATION

We described the design, organization and implementation strategy of a harmonized study to identify a suitable algorithm comprising simple signs that could be feasibly used by frontline workers to accurately predict gestational age. If successful, this method comprising simple signs that do not require intense clinical training to assess, will help health workers in lower level facilities to identify preterm births in these settings where the results will be most relevant (South Asia and sub–Saharan Africa). The results of this study will contribute to mechanisms of targeting interventions aimed at reducing risk of death and significant disability from complications of preterm birth in LMICs [8,27].

The AMANHI gestational age study has many strengths: it is being conducted in five countries in South Asia and sub–Saharan Africa and has adequate power to yield very precise estimates on the validity of these simpler methods; it is one of the few LMIC studies with early ultrasound dating as the gold standard; it targets the identification of preterm babies at the population/community level with large sample size, using non–clinician research workers rather than health professionals in health facilities. The findings will therefore be feasible to implement programmatically at first level facilities or in communities by frontline workers with the barest minimum of professional clinical training. Since many of these preterm births are born at home [28–30] or in these lower level facilities in many LMICs, equipping this cadre of staff with a tool to identify preterm births will be a significant step towards ensuring survival and well–being of preterm babies because tested interventions can be deployed in time for their care.

Despite these strengths, the study also has potential challenges. First, with many deliveries occurring at home, it may be possible that assessors will not be able to reach babies within the narrow 72–hour window particularly since social seclusion after delivery (where women and the babies are secluded from public for up to 40 days) is pervasive in these settings [31–34]. Second excluding babies who are deemed “unwell” may selectively bias the assessments towards term babies because preterm babies are more likely to be perceived as unwell by families and hence assessments may not be done. If assessments are mainly conducted on term babies, component signs may lose their discriminatory power to identify preterm babies. The study sites are therefore using innovative methods to reach babies immediately after they are born. These methods include providing incentives to families for prompt birth notification, regular phone calls to women around term and increasing frequency of home visits to women whose pregnancies were older than 34 weeks. In some sites, selected nurses within health facilities are trained to conduct full assessments on babies who are apparently unwell and cannot be examined by research workers. These nurses use the same forms being used by the AMANHI research assistants. If babies are truly unwell to undergo the full assessments, a select limited number of physical signs and anthropometric measures are assessed by these nurses.

In conclusion, if successful, the AMANHI gestational age study will make a significant contribution to improving the ability of frontline health workers in LMICs to accurately assess gestational age in order to discriminate preterm from term infants. This will allow for more effective targeting of interventions for preterm babies in order to improve their survival. Although identification of preterm babies is the most crucial step for now in the planning and delivery of interventions, it remains only the first step and must be accompanied by concurrent deployment of tested interventions to reduce mortality and residual disabilities in survivors.
